# Computer Enabled Neuroplasticity Treatment: A Clinical Trial of a Novel Design for Neurofeedback Therapy in Adult ADHD

**DOI:** 10.3389/fnhum.2016.00205

**Published:** 2016-05-09

**Authors:** Benjamin Cowley, Édua Holmström, Kristiina Juurmaa, Levas Kovarskis, Christina M. Krause

**Affiliations:** ^1^BrainWork Research Centre, Finnish Institute of Occupational HealthHelsinki, Finland; ^2^Cognitive Brain Research Unit, Cognitive Science, Institute of Behavioural Sciences, University of HelsinkiHelsinki, Finland; ^3^Formally affiliated with Faculty of Behavioural Sciences, University of HelsinkiHelsinki, Finland

**Keywords:** neurofeedback, attention deficit/hyperactivity disorder, attention deficit disorder, adult, randomized controlled trial, waiting list control, learning curves, learning transfer

## Abstract

**Background:** We report a randomized controlled clinical trial of neurofeedback therapy intervention for ADHD/ADD in adults. We focus on internal mechanics of neurofeedback learning, to elucidate the primary role of cortical self-regulation in neurofeedback. We report initial results; more extensive analysis will follow.

**Methods:** Trial has two phases: intervention and follow-up. The intervention consisted of neurofeedback treatment, including intake and outtake measurements, using a waiting-list control group. Treatment involved ~40 h-long sessions 2–5 times per week. Training involved either theta/beta or sensorimotor-rhythm regimes, adapted by adding a novel “inverse-training” condition to promote self-regulation. Follow-up (ongoing) will consist of self-report and executive function tests.

**Setting:** Intake and outtake measurements were conducted at University of Helsinki. Treatment was administered at partner clinic Mental Capital Care, Helsinki.

**Randomization:** We randomly allocated half the sample then adaptively allocated the remainder to minimize baseline differences in prognostic variables.

**Blinding:** Waiting-list control design meant trial was not blinded.

**Participants:** Fifty-four adult Finnish participants (mean age 36 years; 29 females) were recruited after screening by psychiatric review. Forty-four had ADHD diagnoses, 10 had ADD.

**Measurements:** Symptoms were assessed by computerized attention test (T.O.V.A.) and self-report scales, at intake and outtake. Performance during neurofeedback trials was recorded.

**Results:** Participants were recruited and completed intake measurements during summer 2012, before assignment to treatment and control, September 2012. Outtake measurements ran April-August 2013. After dropouts, 23 treatment and 21 waiting-list participants remained for analysis.

Initial analysis showed that, compared to waiting-list control, neurofeedback promoted improvement of self-reported ADHD symptoms, but did not show transfer of learning to T.O.V.A. Comprehensive analysis will be reported elsewhere.

**Trial Registration:** “Computer Enabled Neuroplasticity Treatment (CENT),” ISRCTN13915109.

## 1. Introduction

Attention Deficit/Hyperactivity Disorder (ADHD) is a neurobiological condition which can strongly affect several areas of life: lower socio-economic status, less satisfaction with employment and marriage, as well as common co-occurrence of conditions like addiction and depression. Epidemiology research has estimated the prevalence of ADHD among adults to be 4.4%. Subtypes of ADHD have been identified including Inattentive (ADHD-I), Hyperactive (ADHD-H) and combined (ADHD-C). However, the nature of the disease and most effective method of treatment are still not well understood.

This open-label clinical trial is a test of neurofeedback (NFB) as a treatment intervention for ADHD or ADD-diagnosed adults^1^. The intervention is based on the NFB training regimes “theta-beta” (TB) and “sensorimotor rhythm” (SMR). These regimes train self-regulation of power in specific bands of the EEG frequency spectrum, through the principle of operant conditioning. The trial also includes the novel addition of a third type of training, “inverse training,” designed to help investigate the relationship between NFB learning performance (the requirement to self-regulate) and the specific effects of each training regime. This follows recent calls for increased focus on the internal mechanics of the NFB process by Gevensleben et al. (2014) and Zuberer et al. (2015), in order to attempt more than just another simple test of efficacy.

We test intervention efficacy in a between-subjects manner using a waiting-list control (WLC) group. The trial thus relies on three different levels of measurements: neurophysiological, cognitive, and behavioral; and examines their relationship across multiple time-points. Transfer of learning is measured by: questionnaires tapping ADHD/ADD symptoms that participants filled out at four time-points during the intervention; and by performance on a continuous performance test. More importantly, given our focus on mechanics, we also test specific effects of the NFB training regimes by analysing within-subjects learning curves (LCs) of the treatment group, which represent how they learn to self-regulate their EEG-band activity.

At the time of writing this is an ongoing two phase trial, where phase 1 included intake measurements, NFB intervention, and outtake (all complete), and phase 2 will include follow-up measurements and WLC treatment opportunity (pending). In addition to the protocol structure, we report an initial group-comparison result from the first phase, showing mixed outcomes for efficacy. This result is primarily included to illustrate the importance of the planned within-subjects analysis for obtaining clear insights from the data gathered.

### 1.1. Attention deficit/hyperactive disorder

Models seeking to explain the cognitive neuropsychological problems associated with ADHD include disturbance of attention, cortical arousal, and executive functions (for review see e.g., Sergeant et al., 2003; Seidman, 2006). However, a meta-analysis by Huang-Pollock and Nigg (2003) discarded the explanatory value of attention, at least in individuals diagnosed with the combined subtype of ADHD. Increasingly, ADHD is not seen as a disorder of attention at all but as a disorder in key aspects of self-regulation and executive functions (Nigg, 2005). One caveat is the growing consensus that the executive function “single deficit” model cannot sufficiently explain ADHD (Nigg, 2005; Pennington, 2005; Sonuga-Barke, 2005). Studies indicate that not all persons with ADHD have executive function deficits — at least as measured by laboratory tests.

Cortical arousal models in ADHD are closely related to the attentional concept of alerting as proposed by Posner and Petersen (1990), reflecting right-lateralized vigilance network with noradrenergic involvement. These models emphasize deficiencies in the early stages of information processing as a result of under-arousal in cortical systems (Sergeant et al., 1999). EEG and ERP findings tend to support this model in that they reveal excess slow-wave activity in adults with ADHD (Bresnahan et al., 1999). Support also comes from consistent findings of deficit in the continuous performance test (CPT) d-prime parameter, which can be considered a consensus index of arousal (Losier et al., 1996). Epstein et al. (2003) found that the d-prime demonstrated very robust relationships to the 18 DSMV-IV ADHD symptoms.

EEG studies suggest that ADHD might stem either from a maturational lag or a developmental deviation (Barry et al., 2003). Maturational lag models require that EEG measures from an individual with ADHD would be considered normal in a younger person, and implies that ADHD adults grow out of their immature EEG activity with increasing age (Mann et al., 1992). Whereas in the developmental deviation model, ADHD is conceptualized as resulting from an abnormality in the functioning of the central nervous system, unlikely to change without targeted intervention.

Longitudinal studies, reviewed by Bresnahan et al. (1999), that followed participants up till adulthood revealed that although there is a significant reduction of slow wave activity in both the ADHD and the control group with increasing age, absolute and relative theta activity remained elevated through adolescence into adulthood (Bresnahan and Barry, 2002). Interestingly, with increasing age, the level of beta activity produced by adults with ADHD was normalized in the frontocentral regions. The most consistent finding from EEG studies of ADHD in adults is increased absolute power in theta, clearly visible in frontocentral areas (Bresnahan et al., 1999; Lazzaro et al., 1999; Clarke et al., 2001). Such findings contradict the maturational lag model, as the difference in slow activity does not disappear with increasing age.

By contrast, the hypo-arousal developmental deviation model originally proposed by Satterfield et al. (1974) has been supported by cerebral blood flow and positron emission tomography studies (Lou et al., 1989; Zametkin et al., 1990). This model proposes that ADHD results from cortical under-arousal, and the observed atypical slow wave activity confirms the existence of altered brain activity among adults with ADHD. Hypo-arousal is thought to correlate with both beta and SMR (sensory motor rhythm, also called low beta), because in normal functioning, increased beta is associated with mental activity, and decreased SMR with physical activity.

Much of the existing research has identified maturational lag or hypo-arousal as the underlying cause of ADHD. Although these models have initiated extensive research, they have failed to clarify the aetiology of the disorder (Bresnahan et al., 1999). Thus, the literature suggests that, at least in adult ADHD, aetiological specificity is lacking; with the consequence that traditional treatments targeted at ADHD as a single disorder are unlikely to be reliable. In contrast, personalized medicine emphasizes heterogeneity within a given disorder, relying on biomarkers or endophenotypes to guide different treatments.

### 1.2. Neurofeedback

NFB, also called EEG biofeedback, is operant conditioning of specific temporal, spatial and frequency features extracted from scalp-recorded electrical potentials (Lubar and Shouse, 1976). Feedback is presented to the treated individual in the form of positive and negative reinforcers (in this study: visual reinforcers) whenever their ongoing EEG features meet or fail to meet a predefined criterion. The aim of NFB is to learn to gain control of those EEG features over time.

Literature supports the efficacy of NFB for children with ADHD (Arns et al., 2009, 2014b; Micoulaud-Franchi et al., 2014). Part of its value is that NFB can be personalized to suit the specific clinical presentation, provided that there is requisite theoretical and observational data to guide the personalization.

NFB has been described as a mechanism that can stimulate cortical arousal and/or regulate cortical oscillations, which in turn may influence such cognitive activity as attention (Vernon et al., 2003). The specific effect has been described variously as following one of two models, termed by Gevensleben et al. (2014) as “conditioning- and-repairing model” vs. “skill-acquisition model.” This implies that the effect of NFB may be to repair a presumed cause of disorder to normalize behavior, or instead may be a tool to enhance cognitive performance (see Gevensleben et al., 2014 for a thorough discussion).

It has been suggested that, besides the neurophysiological aspects of NFB, treatment outcome depends greatly on the subjective involvement of the patient. Calderon and Thompson (2004) have conceptualized biofeedback as a three-step process that consists of

- becoming aware of a physiological response,
- learning to control the response, and
- transferring control of the response to everyday life.

The first two steps of the model — becoming aware and learning to control the electrical activity of the brain — constitute NFB learning. The third step refers to *transfer* of the NFB learning, measured here by performance on a neurocognitive test as well as self-reported ADHD related symptoms.

This study employs two kinds of separate NFB training regimes: theta/beta and sensimotor-rhythm. Additionally, we included a novel “inverse mode” of training, which is a modification of each of these two regimes. Finally, transfer trials during which the patient is given no visual feedback were included toward the end of the trial.

Theta/beta (TB) training regime assumes a theta power that is elevated above normal, and therefore uses an inhibition target for theta power and a reinforcement target for beta power. EEG recording is often at a frontal site. The rationale behind TB training has been described in at least two different ways: as the rectification of cortical hypoarousal (Barry et al., 2003), and as the reinforcement of working memory (Vernon et al., 2003).

Sensimotor-rhythm (SMR) training regime reinforces beta power, usually low or mid beta, often with an inhibition target for theta. The site is above the sensorimotor strip, often lateral, such that the beta oscillations correspond to the sensorimotor rhythm. The rationale for SMR training has been proposed as either facilitating attention (Vernon et al., 2003), or the improvement of sleep through an increase in beta spindles, with concomitant effects on cognitive function (Arns et al., 2014a).

It is important to note, that NFB learning is anchored in two scientific theories, but occurrence of NFB learning as such tests only one of these. On the one hand, NFB learning relies on the cortical arousal model of ADHD that emphasizes under-arousal in the cortical systems with excess slow wave activity affecting information processing (Sergeant et al., 1999; Barry et al., 2003). Based on this model, the NFB training aims at increasing fast-wave activity (in this study: SMR and beta bands) and decreasing slow-wave activity (in this study: theta) (Barry et al., 2003). However, NFB learning as such does not test whether the underlying problem of ADHD is under-arousal; what it does test is the operant conditioning of EEG activity. NFB learning is conceptualized in terms of changes in the amount of time a patient manages to move his/her EEG features in the required direction during training sessions as a result of learning to self-regulate cortical oscillations. Zuberer et al. (2015) argue that NFB outcomes should be tested by examination of the learning curves.

Thus, it is clear that, although aetiological and thus clinical specificity for ADHD is lacking, all NFB treatment regimes share a common goal of promoting self-regulation. On the other hand, some more modern NFB regimes have a more explicit approach to self-regulation than their older cousins. For example, Slow Cortical Potentials (SCP) training uses two opposed cortical regulation targets (Mayer et al., 2013), to be trained in random consecutive order. The two most common NFB training regimes TB and SMR do not include such an explicit set of counter-poised targets to induce self-regulation, relying instead on a single target of reinforcement/inhibition, which is trained repeatedly.

The target of SCP training is the Contingent Negative Variation (CNV) Event Related Potential, which Mayer et al. defined as a slow negative shift over central sites that develops following the presentation of warning stimulus while expecting an imperative stimulus that requires a response (Mayer et al., 2015). Thus, while SCP directly addresses self-regulation, it does so only for a single correlate of attention. Other cortical correlates of attention processes are addressed by other NFB training regimes, e.g., cortical hypo-arousal in TB, or spontaneous motor activation in SMR. However, these training regimes have no specific component designed to promote self-regulation.

Therefore, to the standard TB and SMR training regimes we have introduced a mode of “inverse training” (denoted iTB and iSMR), in order to explore the effect of adding an SCP-like approach to these unidirectional training regimes. This takes the form of an extra target in each regime, where the reinforcer/inhibitor is the exact opposite of the norm (see Methods).

TB and SMR training regimes are based on sub-second frequency-band features, so they are not directly comparable with SCP which feeds back the time domain DC component. However, from our point of view, there are at least three motivating reasons to test the “inverse training” self-regulatory modes of TB and SMR.

First, the neurological effect of TB and SMR remains unclear, due to the competing explanatory models. This study will not lay all questions to rest, but it does pursue a novel line of inquiry. Second, “inverse training” should aid the subjective experience of self-regulation, as it does in SCP; after all from the clinical point of view, NFB training in any training regime relies on the patient's own “mental strategy,” reinforced by feedback-free transfer trials. Third, combining the above two issues, the participants have the opportunity to learn the experiential correlate of the inverse neural state, and thus learn to be able to activate OR deactivate cortical resources at will. If we accept, for example, that TB trains the activation of cortical arousal, then the experiential correlate of inverse TB (iTB) might be more appropriate when the individual needs to enter a state of calm reflection. Similarly, SMR implies activation of the sensorimotor strip, which in turn implies quietude of bodily motor-neuron activity; however inverse SMR (iSMR) might be more appropriate when particular task activity calls for so-called kinaesthetic intelligence. This conceptualization of the process would follow the “skill-acquisition” model of Gevensleben et al. (2014). That is, the patient would gain a tool to enhance cognitive performance, as opposed (or in addition) to repairing a presumed cause of disorder.

Although earlier work (Lubar and Shouse, 1976; Monastra et al., 2005), including meta-analysis by Snyder and Hall (2006), has shown support for a single-trait model of ADHD (an elevated theta-beta ratio), others have argued that research results and clinical application should be interpreted with more regard for variability of individuals (Arns et al., 2008). Hammond (2010) goes into this issue in detail, illustrating the heterogeneity in quantitative EEG (qEEG) patterns associated with symptoms and discussing the requirements and need for qEEG analysis guided by normative databases. Johnstone et al. (2005) provided a review of such databases, along with a review of qEEG profiles, which are manifestations seen between genome and behavioral that they term “intermediate” EEG endophenotypes. They called for QEEG endophenotype-guided NFB treatments to provide non-pharmacological interventions to help the subgroup of non-responders to traditional treatments, or complement traditional treatments in certain cases.

Especially in adults, who are subject to maturation effects across a broad age range, ADHD is a heterogeneous disorder with an uncertain treatment situation. In other words, some might have executive function deficits and might possibly benefit from TB over the prefrontal cortex; while some might benefit more from the characteristic behavioral correlate of SMR, that is, immobility as well as reduction of muscular tension (Chase and Harper, 1971; Howe and Sterman, 1972), thus facilitating the self-regulation of attention through mechanisms similar to mindfulness meditation (Zylowska et al., 2008). For this reason, in this study TB and SMR training regimes are assigned in a personalized fashion based on EEG spectral profiles (see Methods).

#### 1.2.1. Outline

In the rest of this paper, following the CONSORT guidelines, we first document the Methods and design of the trial, including participant criteria, intervention details, objectives, outcome measures, sample size calculation, randomization procedure and other allocation details, plus statistical analysis. Next, we provide existing results from the trial as it stands, primarily regarding how the treatment stage was run, along with preliminary analyses of group comparison outcomes. Finally we discuss issues arising from the trial design and implementation, as well as the implications of the preliminary analyses and future work.

## 2. Methods/design

### 2.1. Participants

Inclusion criteria were scores on Adult ADHD Self Report Scale (ASRS) (Kessler et al., 2005), and Brown -ADHD scale (BADDS) (Brown, 1996) indicating presence of ADHD, as well as:

- pre-existing diagnosis of ADHD or ADD,
- nonexistence of neurological diagnoses,
- age 18–60 years,
- IQ score >80 measured by a qualified psychologist using WAIS IV (Wechsler, 2008)

Exclusion criteria included extreme outlier scores in the scales of

- Generalized Anxiety Disorder (Spitzer et al., 2006),
- Beck Depression Inventory (Beck et al., 1996),
- Alcohol Use Disorders Identification Test (Saunders et al., 1993),
- the Mood Disorder Questionnaire (Hirschfeld et al., 2000),
- test of prodromal symptoms of psychosis (Heinimaa et al., 2003), and
- the Dissociative experiences scale (Liebowitz, 1992) for dissociative symptoms.

Thresholds for exclusion were not fixed but at the discretion of the consulting psychiatrist. Use of medication for ADHD was not an exclusion criterion but participants were asked not to make changes in medication during the time of the training. Informed consent was obtained from each subject in accordance with the Declaration of Helsinki.

Self-report tests were distributed and submitted by mail. Psychiatric consultations were performed at the private practice of the psychiatrist; IQ testing was performed at the testing room of the University of Helsinki (UoH) Institute of Behavioral Sciences (IBS); both in central Helsinki.

#### 2.1.1. Ethical approval

Written informed consent for participation was obtained from all participants before entering the study. The protocol followed the Declaration of Helsinki for the rights of the participants and the procedures of the study. An ethical approval of the present research protocol for all participants was obtained from The Ethical Committee of the Hospital District of Helsinki and Uusimaa, 28/03/2012, 621/1999, 24 §. Participants were not remunerated.

#### 2.1.2. Clinic of treatment

The clinic for intervention sessions was required to be centrally located in Helsinki, to be staffed by licensed psychiatrist in case of emergencies, and to have recognition by the Association for Finnish Work. Technicians were required to have a primary degree in a discipline related to human psychology; they were also required to take a 3-month training course provided at the UoH based on principles of the Biofeedback Certification International Alliance (BCIA).

### 2.2. Intervention

The experimental treatment was a novel neurofeedback (NFB) intervention, based on the well-known operant conditioning NFB training regimes “theta-beta” (TB) and “sensorimotor rhythm” (SMR); with the novel addition of a self-regulatory component designed to address the heterogeneity and aetiological uncertainty of ADHD in the adult population. The comparator was a WLC group. The WLC design places a randomly assigned control group on hiatus, while the active treatment is applied to the randomly assigned treatment group. The WLC group should receive treatment after the follow-up assessment at 24 months post-treatment, without experimental oversight.

Participants who volunteered in response to advertisements were recruited at time T0. Contact with the psychiatrist and psychologist for screening followed at time T1.

Successfully screened participants were taken to the intake measurement at time T2, where they performed the T.O.V.A. test along with eyes-open and closed baselines, while scalp EEG was recorded. The individual alpha peak frequency (IAPF) of each participant was estimated from band power analysis of eye-opened and eye-closed baseline conditions (Lansbergen et al., 2011). The boundaries of each EEG frequency band for each participant are defined with respect to IAPF, e.g., theta is IAPF × 0.4 to IAPF × 0.6.

After randomization between treatment and WLC groups at time T3, we assigned participants in the NFB treatment group to either TB or SMR training based on their IAPF-adjusted theta/beta ratio. Those with theta/beta ratio >1 (*n* = 9) received reinforcement for simultaneous increase in beta and decrease in theta (over power estimated from per-session baseline) at electrode Fz. The rest (*n* = 16) got reinforcement for increase in SMR and decrease in theta at electrode C4. Band powers within the NFB training regimes are adjusted by IAPF.

Treatment was administered using the following hardware and software set up. The EEG amplifier was the Enobio ambulatory device (Neuroelectrics SL, Barcelona)^2^, with streaming Bluetooth connection to standard Windows 8 desktop computers. The software was developed within the project, as described in Cowley et al. (2016). Briefly, the system is based on OpenViBE signal acquisition framework^3^, with a Qt frontend, and is available open source^4^.

NFB interventions were standardized by scheduling of the training sessions: session duration was fixed; and training blocks per session, sessions per week, timing of the break from training, and total duration of training were all constrained to equalize the intervention. At time T4, treatment group participants began their treatment by being briefed about all aspects of the NFB training regimes, e.g., length, frequency, purpose. Finally for the first phase, outcome measures were taken at time T5, when all participants in the treatment group had completed 40 sessions NFB.

In the first phase the care providers were monitored by both the lead researcher and responsible psychiatrist on separate occasions, with interviews to ascertain their self-assessment of performance. Both care providers and patients were given self-assessment questionnaires to describe their working relationships.

The second phase of the trial is ongoing at time of writing. Beginning with re-recruitment at T6, phase two consists of a follow-up measurement for all, and treatment option for WLC participants. Follow-up measurements include the ASRS and BADDS self-reports. Treatment for the WLC group will not be NFB, but will consist of a game-like computerized attention training intervention, without concurrent recording of EEG.

### 2.3. Objectives

Our RCT research questions (RQs) follow Calderon and Thompson (2004), since we first examine NFB learning “within subjects,” and second examine the transfer of NFB learning comparing the treatment group to a WLC group. Within this paper, we report only first-stage analysis, namely the comparison between groups addressed by H2a and H3c below (see Results).

#### 2.3.1. Learning in NFB

The NFB learning metric reflects the proportion of time during training when EEG signals are in the target state; the “learning curve” is thus characterized by a signal evolving over blocks and sessions of training. The shapes or slopes of participants LCs are rarely reported in the NFB literature: analysis tends to focus on transfer outcomes compared to a control group. However, clinical observations commonly indicate that learning occurs. Also, NFB learning in this study was manipulated with the addition of the “inverse-training” mode. The LCs resulting from normal, inverse and transfer training blocks should each be slope-positive, because they each require a similar act of concentration which the participants are practicing throughout training. Finally, the profile of the LCs over sessions which combine all training types should be slope-positive, because training with counter-poised targets increases the need to self-regulate. Thus, we propose the following hypotheses:

- H1a: “normal” NFB training results in positive-slope LCs.
- H1b: “inverse” NFB training results in positive-slope LCs.
- H1c: “transfer” NFB training results in positive-slope LCs.

#### 2.3.2. Transfer: attention test and self-reported symptoms

Due to meta-analyses that find that NFB is efficacious for reduction of inattention (Arns et al., 2009), transfer of learning is expected to result in more improvement-over-baseline of the treatment group, compared with a control group, at the continuous performance test (CPT) Test Of Variables of Attention (T.O.V.A.) applied before and after training. Furthermore, those participants who perform better in baseline T.O.V.A. (lower scores), are expected to learn quicker during the NFB training.

- H2a: the treatment group will achieve better T.O.V.A. performance, and improve more after training, than the WLC.
- H2b: a better baseline T.O.V.A. score will predict better baseline NFB performance and better NFB learning.

Severity of subjective symptoms of ADHD/ADD should be reduced by the transfer of NFB learning to the ability to self-regulate. We also expect this effect to be dose-dependent, such that participants with better NFB performance (steeper positive slope) should present a higher rate of change in reported symptoms (steeper negative slope). Finally, the treatment group is expected to report fewer symptoms than the control group in the outcome measurement.

- H3a: NFB training will result in a negative linear trend in reported ADHD/ADD symptoms.
- H3b: the NFB LC profile will correlate with reported ADHD/ADD symptoms.
- H3c: the treatment group will report greater improvements in ADHD/ADD symptoms than the WLC.

### 2.4. Outcomes

Primary outcome measures include learning curve assessment, T.O.V.A., ASRS, and Digit span. These measures fall into two categories: (1) between-group comparison of NFB and WLC groups; and (2) within-subjects tests for treatment group. Primary measures are all comparative, truly experimental, and hypothesis-driven.

Secondary outcome measures include pre- and post-treatment vigilance measurement with an EEG protocol (Olbrich et al., 2012); also per-session self-report of circadian patterns, mood, excitement, effort and frustration; and the Pittsburgh Sleep Quality Index (PSQI) administered pre-, post- and at two intermediate points during treatment. These measures were taken to explore the additional research question of the relationship between sleep quality and NFB performance.

Additional methods taken to assess the quality of measurements include

- placebo expectation reports (Borkovec and Sibrava, 2005) asked before and at halfway through the treatment,
- the novel participant-technician interaction questionnaire, designed to assess the participants' experience of their interaction with the technician. Based on the guidelines of the “Framework for Measuring Impact”^5^, we created what they term “specifically developed questions.” This option was especially appropriate given the requirement to translate any existing measure, which would limit validity. The questionnaire was a set of ten simple questions such as “has my trainer supported me during my neurofeedback training?” Responses were made on a six-point Likert-scale from “0-not at all” to “5-enough,″ for a scoring range of 0–50.

### 2.5. Sample size

The power calculation of *N* = 60 was based on an estimated effect size for neurofeedback of 0.9, alpha at 0.05 and Power at 0.95 (for an independent samples *t*-test, estimate based on studies including Egner and Gruzelier, 2004; Rossiter, 2004a,b; Arns et al., 2009). Recent work has shown an effect size of 0.73 for a purely adult population (Mayer et al., 2012), but we can still maintain *N* = 60 by assuming power = 0.85, since this is equivalent to an phase 1 trial where the motivation is greater to minimize Type I error than Type II. This also contributes to our motivation to use a WLC along with the principle that NFB is closer in nature to a behavioral intervention than a drug trial, and thus double-blinding is unwarranted and unethical.

### 2.6. Randomization procedure

We assign patients to test or control groups using a procedure that controls for selection bias, and known and unknown sources of external variance due to prognostic variables; the procedure is based on combined randomization and adaptive allocation.

Simple randomization prevents biased selection and meets the assumptions of uniform assignment probability made in standard inferential procedures. However, it does not guarantee equal group sizes and can lead to baseline imbalance in prognostic variables such as age, gender or disease severity. Equal group sizes can be obtained using blocking, while baseline imbalance can be helped by using blocking stratified over prognostic variables, although the number of variables which can be used is small. This limitation is surmounted by adaptive allocation, exemplified by minimization methods: these are not strictly random but allow tight control of balance of multiple prognostic variables (see e.g., Roberts and Torgerson, 1998 on methodological issues).

In their review of recommendations of assignment method, (Scott et al., 2002, p. 671) found a general support for minimization with a random element in smaller trials. Our approach follows: X% random blocking followed by 100-X% minimization (0<X<100). The algorithm is:

1. Simple blocked random allocation of X% patients (of those currently recruited).
2. For the next 100−X% of patients, reassign patients based on minimization.
3. Using a distance measure, make the assignment that results in the smaller inter-group distance.
4. For every new assignment decrement the number of possible new assignments to that group, to maintain equal group sizes.

Assignment was balanced over age, sex, education, IQ, diagnosis (ADHD vs. ADD), comorbities from diagnosis, comorbities from administered scales, and ASRS subtype score, and tested between groups to show no statistically significant differences (see Table 1 below). The same tests returned null when run after any change in relative group composition, due to e.g., drop-outs. Thus, groups did not differ in terms of symptom severity, diagnosis, IQ or demographic features at assignment.

**Table 1:** **Demographic and clinical characteristics for NFB and WLC groups**.

		**Treatment**	**Control**	**Statistical testing**		
		***N = 25***	***N = 29***	***t***	***df***	***p***
Age	Mean	35.72	36.45	−0.259	52	0.797
	Std. Dev.	9.66	10.86			
WAIS-IV: VCI	Mean	113.68	113.59	0.035	52	0.972
	Std. Dev.	10.33	9.37			
WAIS-IV: POI	Mean	113.44	112.69	0.194	52	0.847
	Std. Dev.	11.87	15.87			
				χ^2^	***df***	***p***
Gender	Female	14 (56%)	15 (52%)	0.99	1	0.753
	Male	11 (44%)	14 (48%)			
Education	Primary	6 (24%)	5 (17%)	0.470	2	0.791
	Secondary	15 (60%)	18 (62%			
	Tertiary	4 (16%)	6 (21%)			
ADHD/ADD	ADHD	21 (84%)	23 (79%)	0.196	1	0.658
	ADD	4 (16%)	6 (21%)			

Given that the initial allocation of patients is random, further non-random selections will also be random at the population level. Even if the minimization assignments cannot be considered random, the overall assignment retains a proportional amount of randomness equivalent to X, similar to more complex biased-coin approaches. In this trial X was set equal to 50.

The most important caveat of the approach is that variables used in minimization must be included as covariates during analysis, to avoid potentially misleading results.

Technicians must be assigned to treatment and control group by recruitment on-demand, as the group treatment phases were separated in time.

#### 2.6.1. Allocation concealment

Participants were randomly assigned by use of computerized algorithm detailed above, at one time, directly after all intake measurements and before the start of NFB treatment.

#### 2.6.2. Implementation

The allocation algorithm was designed and implemented by the lead researcher; the direct contact with participants for enrolment and assignment was handled by a technician working for the MCC clinic.

#### 2.6.3. Blinding

Due to the fact that the trial used waiting list for the control group, assessment was not blinded. Thus, after assignment, all participants and researchers/technicians had access to the assignment information. As mentioned above, since NFB is closer in nature to a behavioral intervention than a drug trial, double-blinding would be unwarranted and unethical.

### 2.7. Statistical methods

Independent variables for group comparison include NFB training regime (TB vs. SMR), participant age and gender, and assigned group (treatment vs. WLC group). Dependent variables (DVs) for group comparison include the T.O.V.A. and the ASRS self-report.

T.O.V.A. variables, based on response times (RT) and error rates, include RT variability (RTV) indicating consistency; mean RT; Omission errors (OM) indicating inattention; Commission errors (COM) indicating impulsivity; as well as the D-prime score. D-prime is described as a measure of “perceptual sensitivity” and has been suggested as an index of arousal (Losier et al., 1996). T.O.V.A. variables are standardized in the analysis.

To evaluate the effect of NFB on T.O.V.A., we created five new variables by subtracting the baseline scores from the outcome measurement scores: RTV-change, mean RT-change, OM-change, COM-change, and D-prime-change. These T.O.V.A. change scores are subsequently compared for the treatment group and the WLC, using independent samples *t*-test. The Levene's test showed that the variances for the two groups were similar. Consequently, the independent samples *t*-test was run with equal variances assumed.

MANOVA was subsequently used to evaluate the effect of NFB training, compared to the WLC, in the outcome measures on the five dependent variables of T.O.V.A.

ASRS consists of 18 items tapping the frequency of recent DSM-IV criterion symptoms of adult ADHD, including a scale for Inattention (IA, max 36 points) and a scale for Hyperactivity-Impulsivity (HI, max 36 points). We calculated differences of scores between baseline and outcome measurements to create IA-change and HI-change scores. These difference scores are subsequently compared for the NFB and the control group, using independent samples *t*-test.

The Levene's test showed that the variances in IA-change were statistically significantly different in the two groups (*F* = 4.36, *p* < 0.05). As a result, the independent samples *t*-test for IA-change was run with equal variances not assumed. The opposite was the case for the variable HI-change.

In all our random coefficient models the intercept and slope are separately estimated for each participant. That is, the coefficients are estimated for each participant for the linear regression equation as follows: Score = Intercept + B (Session).

All participants were treated with NFB at a single center. All technicians were equivalently trained and capable; therefore clustering of participants per technician was based simply on scheduling logistics.

## 3. Results

Results primarily describe the specific details of the intervention implementation. Also, as stated, a preliminary between-subjects comparison was performed after phase one to assess intervention efficacy under a conventional analysis model. Thus, we report the two straightforward pre- to post-treatment outcome measures which are comparable between-groups: T.O.V.A. and ASRS. The ambiguous results of this conventional approach, especially in an unblinded context, supports the motivation to extend the analysis with LC modeling. We therefore include these results in the protocol report because they constitute an informative part of the trial design going into the second phase.

### 3.1. Participant flow

Eighty-two adults were recruited through cooperating clinics Mental Capital Care, Neuromental and YTHS; also by newspaper advertisement and posting to online forums for the Helsinki-based ADHD society of Finland. Of this, 19 dropped out of the trial before completing the screening process, due to various issues.

Sixty-three participants were screened by a psychiatrist and a psychologist prior to the training, resulting in nine participants screened out of the trial due to one or more failures to meet the criteria. The remainder (*n* = 54) consisted of 29 females, 25 males, mean age 36 std.dev. 10 years, with 44 ADHD and 10 ADD diagnoses.

**Figure 1:**
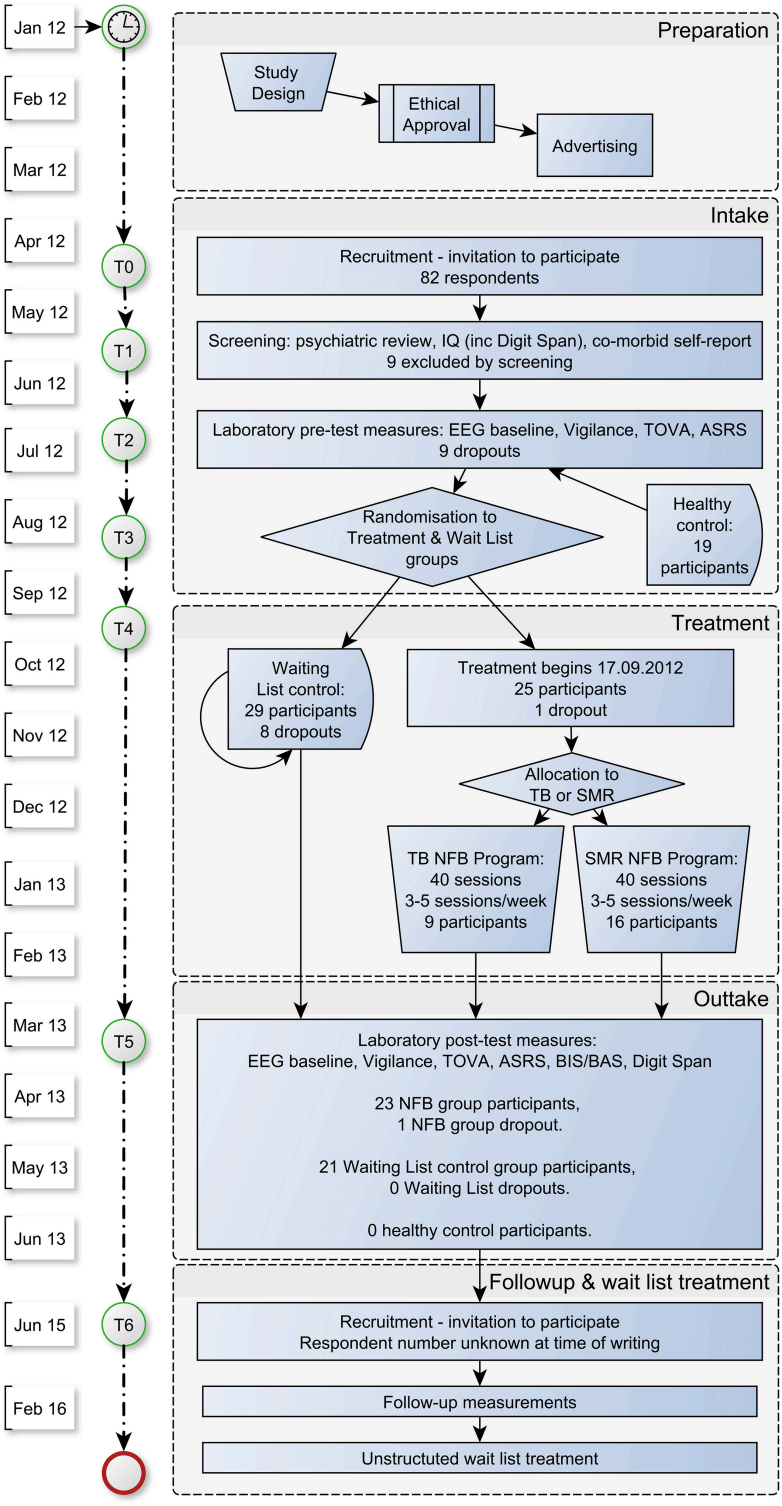
**Flow chart of assessments and treatments in the CENT trial**.

Participants were split equally between treatment (*n* = 27) and control groups (*n* = 27); however two switched from treatment group (final *n* = 25) to control group (final *n* = 29) for personal reasons. From these assignments, eight dropped out from the WLC group (including one participant whose pre-test measurement data was then deleted by request), and two from the treatment group. Thus, 23 treatment group, and 21 WLC group cases (total *n* = 44) were available for analysis. The trial progression is shown in detail in Figure 1.

Out of those who completed NFB, five participants did not complete treatment on the pre-defined schedule, of which three exceeded by more than a week. Delays were due to personal reasons, causing a number of cancelations of scheduled sessions, which is a regularly observed phenomenon in this diagnostic group.

#### 3.1.1. Implementation of intervention

In practice, NFB training consisted of ~40 sessions (range: 38–41) during 2–4 months. There was a mid-training pause of nominally 2 weeks. Patients came to the sessions 2–5 times a week. One session lasted ~1 h, subdivided into self-report of mood, excitement, hours slept and hours awake; electrode attachment; baseline measurement; 5–7 units of 5 min NFB trials; and debrief including self-report of effort and frustration. During each session, patients played different NFB “game” trials during which they got immediate visual reinforcement for classifier-matching states in their EEG. The scores per game trial are baseline-adjusted and averaged per session to form characteristic LCs. The content and purpose of the training sessions followed a phased timeline:

1. Tutorial stage, for becoming accustomed to NFB, two practice sessions: participants were given normal NFB trials with baseline thresholds adjusted by a constant factor to make the training easier;
2. Beginner stage, for NFB training, 18 sessions up to halfway break: normal NFB with non-adjusted baseline thresholds;
3. Intermediate stage, for learning to self-regulate, ten sessions from half-way to session 30: normal training blocks were gradually reduced in number to half per session, and inverse training blocks introduced in their place;
4. Expert stage, for transfer training, ten sessions until session 40: as Intermediate stage, but also with one to two “transfer” trials with no feedback stimuli.

#### 3.1.2. Recruitment

As shown in Figure 1, participants were recruited in May/June 2012, with intake measurements during July/August 2012. Randomization took place in early September; treatment began September 17, 2012. Outtake measurements ran from April until August 2013.

Follow-up measurements are planned for start of 2016.

### 3.2. Baseline data

The participants who began the trial (*n* = 54, 29 females) had mean age 36 years (std.dev. 10 years), with 44 ADHD and 10 ADD diagnoses. The characteristics per group are shown below in Table 1.

All intervention sessions were performed at the Mental Capital Care (MCC) clinic premises in Helsinki, which met all requirements described above. Technicians were recruited to administer the NFB from IBS and MCC; they were trained at UOH over a 3 month period; four of the team also attended the Biofeedback Certification International Alliance (BCIA)-accredited introductory course at the Brainclinics education and treatment center, Netherlands.

#### 3.2.1. Numbers analyzed

The analysis included 23 participants in the treatment group, and 21 participants in the WLC group. Given that dropouts were not analyzed, this analysis was not by intention-to-treat.

### 3.3. Outcomes and estimation

For H2a we find no support after analysis of the five T.O.V.A. indexes; Table 2 shows their mean differences between baseline and post-training. Changes in these variables were not significantly different for the NFB group than for the WLC. Results of the MANOVA at the outcome measurement revealed that on the Wilks' Lambda the difference in means between the NFB and WLC did not reach significance [*F*_(5, 38)_ = 0.45, *p* >0.05]. Thus, NFB training had no significant effect on the 5 indexes of T.O.V.A. at the end of the intervention.

**Table 2:** **Groups statistics for RTV-change, mean RT-change, OM-change, COM-change and D-prime-change scores from baseline to outcome**.

	***N***	**Mean**	**Std**.	***t***	***df***	***p***
RTV-change				0.343	42	0.739
NFB group	23	−5.56	40.13			
WLC	21	−9.44	34.15			
Mean RT-change				0.132	42	0.896
NFB group	23	−3.37	16.23			
WLC	21	−3.94	11.85			
OM-change				1.19	39	0.240
NFB group	23	10.10	72.73			
WLC	21	−46.25	198.84			
COM-change				0.517	42	0.608
NFB group	23	6.19	27.49			
WLC	21	1.81	28.67			
D-prime-change				−0.016	42	0.987
NFB group	23	1.50	45.30			
WLC	21	1.72	44.73			

Table 3 presents the mean difference of the two indexes between baseline and post-training and the results of the independent sample *t*-tests comparing IA-change and HI-change between NFB group and WLC. The NFB group presented a higher reduction of inattention symptoms than the WLC *t*_(36.03)_ = −2.14, *p* < 0.05. Similarly, while the NFB group presented a reduction of HI symptoms from baseline to post-training, the WLC presented an increase in HI symptoms *t*_(44)_ = −2.42, *p* < 0.05. Thus, we find statistically significant support for H3c. That is, the treatment group reported greater improvements in ADHD/ADD symptoms than the WLC.

**Table 3:** **Groups statistics for IA-change and HI-change scores**.

	***N***	**Mean**	**Std**	***t***	***df***	***p***
IA-change				−2.14	36.03	0.039
NFB group	25	−1.2	2.17			
WLC	21	−0.14	1.06			
HI-change				−2.42	44	0.020
NFB group	25	−1.08	2.31			
WLC	21	0.38	1.65			

A more detailed analysis concerning the rest of the hypotheses will follow in a separate paper. As treating the hypotheses H1a-b, H2b, and H3a-b requires substantial additional methodological reporting, addressing them does not fit the scope of a clinical trial report.

#### 3.3.1. Adverse effects

No adverse effects were observed in the treatment group. Further investigation of this question is planned for follow-up, using the state-oriented self-report items described above.

## 4. Discussion

### 4.1. Interpretation

Regarding the relationship between NFB learning and performance in the continuous performance test, H2a proposed that the NFB group will achieve better T.O.V.A. performance, and improve more after training, than the WLC. Results of this study did not find evidence for such transfer. Patients participating in the NFB training did not perform better than WLC on the 5 indexes of T.O.V.A. in the outcome measurement. This result can be interpreted in at least two ways. On the one hand, it can be a sign of the all too common problem of transfer of training (Green and Bavelier, 2012).

Lack of transfer is one of the most important of the several key obstacles pertaining to the effect of NFB trainings (Gazzaniga, 2009, p. 94). Because brain plasticity is highly task specific, training in a specific task shows little or no improvement on related tasks. On the other hand, the results of this study can mean that NF learning bears no relationship to performance on any indexes of the T.O.V.A. test. This would contradict the findings of Losier et al. (1996) who considered the D-prime index of T.O.V.A. a consensus index of arousal, which is, in turn, assumed to be a manifestation of excessive slow wave brain waves in ADHD patients (Barry et al., 2003).

H3c suggested that the NFB group will report greater improvements in ADHD symptoms than the WLC. Results show the change of IA was significant. Patients did perceive a reduction of inattention symptoms over the course of the training. Furthermore, this perceived reduction of inattention symptoms differed significantly from the perceived reduction of inattention symptoms of the control group. This supports the meta-analysis by Arns et al. (2009) concluding that NFB has large effect sizes on inattention. In the index of HI, no negative linear trend was found. Interestingly however, patients in the NFB group did perceive a significantly larger reduction of these symptoms over the course of the training than the control group.

It might be that the training indeed resulted in some reduction of IA and HI symptoms. This interpretation gets support from theories claiming that ADHD is, in effect, pathology of executive functions that cannot be tapped by neurocognitive tests, but can instead be measured by self-reported questionnaires (Rabbitt, 1997; Brown, 2009, pp. 81–116). Alternatively, the results can also be interpreted as an example of the so called Hawthorne effect (Green and Bavelier, 2012). Establishing the presence of experience-dependent learning effects is not always straightforward. It is well documented, that individuals who take an active interest in their performance tend to improve more, or evaluate their improvement more positively. The Hawthorne effect can lead to powerful subjective improvements that have little to do with the specific cognitive training regimen being studied reflecting motivational factors instead.

If the training caused the decreased symptoms, a higher rate of learning should have a relationship to the rate of ADHD symptom decrease under the training. Therefore further analyses of this trial will examine the relationship between NFB LCs and the trend of self-reported ADHD symptoms.

Also, the different learning performance levels across the group should reflect different long term effects, to be measured during the second phase in a within-subjects analysis. This contrasts with the effect from the interaction-derived placebo which is relatively constant for all participants (per group), and presumably “fades away” quickly after the phase one intervention. Thus, second phase measurements will be analyzed with respect to NFB LCs.

Though this is a relatively small study, we believe the analysis of learning curve questions is a novel and useful contribution. As noted by Arns (personal communication, 2012):

“In any Neurofeedback study it is very important to track and have an indication of ‘learning.’ If neurofeedback fails to demonstrate a clinical effect and there is no indication that learning actually took place, one can't draw any conclusions about neurofeedback. In an analogy, if one employs operant conditioning to [teach] a rat to press a lever, and the rat does not learn to press the lever, then it is incorrect to conclude that ‘operant conditioning’ does not work. This means that maybe the operant conditioning procedure was not implemented effectively. The same applies to neurofeedback, and this is further illustrated by the study from Roger DeBeuss. His study employed sub-optimal parameters e.g., auto-thresholding, ‘game’ feedback and an ‘unconventional’ training regime (engagement index) making it likely harder to learn. On the group level they found no effects of neurofeedback in ADHD, however, when separating learners from non-learners based on session data they did find an effect.”

Questions of efficacy on the other hand are still a matter of controversy in the literature. WLC controls are not accepted as sufficient evidence by some. Double blind control through sham NFB was not chosen for this study because of the discussed lack of general understanding of ADHD. This lack, combined with the extremely contingent nature of NFB which depends heavily on non-specific aspects of treatment, implies that even if a double blind RCT showed large effect for NFB the causal mechanisms would still not be clear. A true resolution to this issue is probably only possible by running the kind of large sham NFB RCT called for by others; we choose instead to side-step this debate and focus on questions of internal comparisons within the method.

There is no blinding in this open-label WLC study, except at random assignment to groups. Other control paradigms may be preferred in pharmacological interventions; however there are a number of arguments in support of WLC, in the context of NFB. The WLC is a minimum viable control for non-specific effects of history, maturation, repeated testing, instrument drift, statistical regression, selection bias, and population inhomogeneity effects (Mohr et al., 2009). There is no control for non-specific or placebo effects; however this is still a valid experimental control design. Among other things, WLC controls for expectation and attention (Hawthorne) effects, whereby the notion that at some future point treatment will be provided (and life will improve) is by itself able to produce improvement. Additionally, longitudinal (rather than parallel) designs control for maturation, regression to the mean, instrument drift and practice effects; also time threats to validity (the same effect occurring 2 years in a row in different samples rules out external non-seasonal temporal causes for the effect. Seasonal causes with a WLC group should be ruled out by staggered application i.e., 2nd treatment starting in spring).

Technicians can be considered equally skilled and expert. All began as neurofeedback novices before the trial. Qualification history was varied. Due to availability, only some began their training with attendance at the Biofeedback Certification International Alliance (BCIA)-accredited introductory course at the Brainclinics education and treatment center, Netherlands. However, all five then shared 3 months training at UoH premises, including extensive peer review work, which helped to disseminate and pool the knowledge across the group.

### 4.2. Generalizability

External validity asks the question of “generalizability”: to what populations, settings, treatment variables and measurement variables can this effect be generalized? While the question of external validity is never completely answerable, it is of particular interest for intervention research (Campbell et al., 1966, pp. 171–246). Campbell et al. (1966) note that there is a recurrent reluctance among researchers to accept Hume's truism that induction or generalization is never fully justified logically. While the problems of internal validity are solvable within the limits of the logic of probability statistics, the problems of external validity are not logically solvable in any neat, conclusive way (Campbell et al., 1966, p. 17). Generalization always involves extrapolation into a realm not represented in one's sample. Here, the issue of sample bias is of importance. If an experimental study is conducted with voluntary patients from a given district, they might have characteristics that cause the experimental treatment to be more effective than it would be in other populations. However, for ethical reasons, intervention studies are impossible to conduct without the informed consent of the research subjects. It is obvious, that a “true” experimental design is in practice impossible with a fully representative sample of a given country, let alone all ADHD patients in the world. It must be emphasized that the results of an experiment “probe” but do not ″prove″ a theory. An adequate hypothesis is one that has repeatedly survived such probing, but it may always be displaced by a new probe. Many findings in experimental psychology gain generalizability not through the nature of the setting in which they occurred, but through their ability to establish a theory of basic mental processes that are implicated in many tasks.

### 4.3. Overall evidence

After preliminary analysis, the trial did not find evidence for a transfer of learning that was the intended benefit of the intervention. Since the intervention's goal is symptomatic improvement outside the laboratory, the results underline how the transfer-problem limits the potential benefits. Patients did perceive a reduction of Inattention symptoms over the course of training, yet this change was not reflected in better performance in the continuous performance test (T.O.V.A.).

We propose that in the absence of any other evidence, one should consider these self-report results as due to placebo by default. The improvement in self-reported symptoms might not be specifically due to NFB, but due to employment of goal-oriented attention in general. At present, we can not rule out the possibility that the individuals would also report improvement if all other factors were held equal but NFB was swapped for some other exercise that requires concentration. That is, our preliminary results did not support the effectiveness of NFB in alleviating the symptoms of AHDH/ADD.

Nevertheless, keeping in mind the core aim of studying mechanisms and models of NFB, the CENT trial is on track to provide the necessary evidence. As Seidman (2006) suggest, an adequate neuropsychological model of ADHD should utilize measures from multiple domains to be able to encompass subtypes and multiple deficits. Combining neuropsychological, neurophysiological and behavioral measures, we aim toward an evaluation of structure-function relationships in NFB treatment for adult ADHD.

## Author contributions

BC designed and implemented the study and wrote the draft text; EH conducted the statistical analyses and contributed to the draft; KJ and LK contributed to the study design and implementation, and the draft; CK contributed to the study design and the draft.

### Conflict of interest statement

The authors declare that the research was conducted in the absence of any commercial or financial relationships that could be construed as a potential conflict of interest.

## Footnotes

^1^The ICD-10 diagnosis classification system is in use in Finland where the study was conducted. Both ADD and ADHD are used as diagnoses, the former referring to the predominantly inattentive type, as opposed to predominantly hyperactive or combined types.

^2^http://www.neuroelectrics.com/.

^3^http://openvibe.inria.fr/.

^4^https://github.com/CBRUhelsinki/CENTplatform.

^5^http://www.measuringimpact.org/home.

## Abbreviations

ADHD: Attention Deficit/Hyperactivity Disorder
ADD: Attention Deficit Disorder
BCIA: Biofeedback Certification International Alliance
CENT: Computer Enabled Neuroplasticity Treatment
CNV: Contingent Negative Variation
IBS: Institute of Behavioral Science
MCC: Mental Capital Care (clinic)
NFB: neurofeedback
RCT: randomized controlled trial
SCP: Slow Cortical Potentials (neurofeedback training regime)
SMR: SensoriMotor Rhythm (neurofeedback training regime)
TB: Theta/Beta (neurofeedback training regime)
T.O.V.A.: Test Of Variables of Attention
UoH: University of Helsinki
WLC: waiting list control.
